# Challenges in the Interpretation of Dengue Vaccine Trial Results

**DOI:** 10.1371/journal.pntd.0002126

**Published:** 2013-08-29

**Authors:** Isabel Rodriguez-Barraquer, Luis Mier-y-Teran-Romero, Donald S. Burke, Derek A. T. Cummings

**Affiliations:** 1 Department of Epidemiology, Johns Hopkins University, Baltimore, Maryland, United States of America; 2 Nonlinear Systems Dynamics Section, Plasma Physics Division, U.S. Naval Research Laboratory, Washington, D.C., United States of America; 3 University of Pittsburgh Graduate School of Public Health, Pittsburgh, Pennsylvania, United States of America; Pediatric Dengue Vaccine Initiative, United States of America

Several hypotheses have been proposed to explain the unexpected results of the first completed efficacy clinical trial of a vaccine against dengue virus [1]. Based on intention-to-treat analyses, the vaccine was efficacious in reducing the incidence of clinical disease caused by dengue serotypes 1, 3, and 4, but failed to reduce the incidence of dengue-2 (DENV-2). The authors of the study propose potential explanations including an antigenic mismatch between the parental strain of the DENV-2 component and currently circulating DENV-2 viruses in Ratchaburi, an increased role for immunity to nonstructural proteins in DENV-2 that this vaccine does not induce, and a lack of correlation of measured neutralizing antibody and protective immunity [1].

However, we believe that in addition to questioning the immune response elicited by the vaccine, it is important to discuss the interpretability of efficacy results for dengue vaccine trials that are based exclusively on clinical outcomes. The study by Sabchareon measured vaccine efficacy against clinically apparent infection (VE_C_). This is distinct from vaccine efficacy against infection (VE_I_), and potentially a very important distinction.

VE_I_ is a function of the incidence of infection in the control and vaccine groups and measures the vaccine's capacity to induce an immune response that will prevent infection. In contrast, VE_C_ is a function of the incidence of clinical disease in the control and vaccine groups, and depends not only on the incidence of infection, but also on the probability of developing clinical disease after infection. VE_C_ and VE_I_ will only be exchangeable if the probabilities of clinical disease (often described as the symptomatic∶asymptomatic ratio) are equal in the control and vaccine groups.

Dengue disease is caused by four interacting viral serotypes. Risk factors for symptomatic disease have not been fully characterized. There is substantial evidence that preexisting, naturally acquired immunity against a heterologous serotype is a significant risk-factor for the development of clinical disease [2]. Whether vaccine-induced immunity acts in the same way is not clear, though this is possible and plausible [3]. If vaccine-induced immunity does resemble naturally acquired immunity, it clearly has the potential to modify the probability of clinical disease among vaccine recipients and obscure the relationship between VE_C_ and VE_I_. Figure 1 illustrates an example of how a vaccine with high VE_I_ would seem to be ineffective in a trial based only on clinical outcomes.

**Figure 1:**
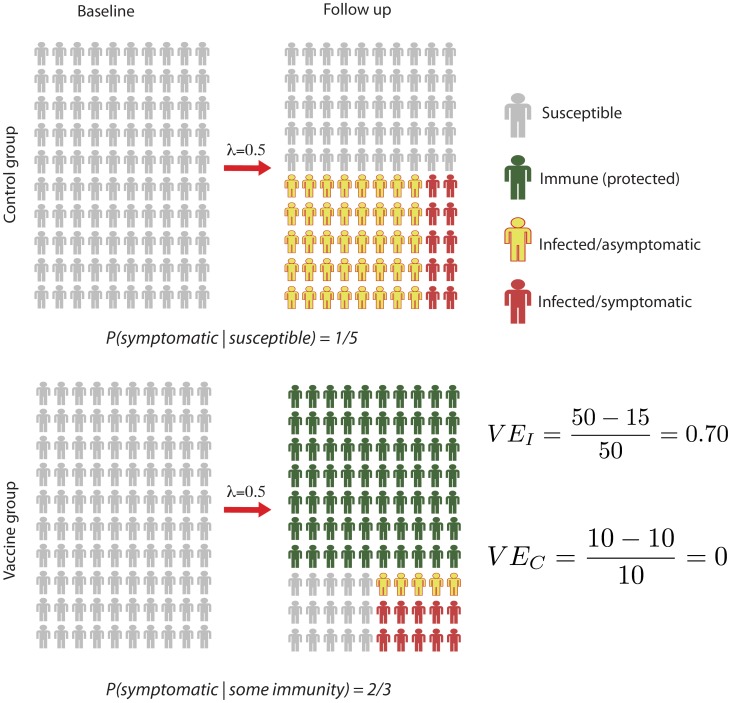
Figure showing the lack of agreement that might exist between VE_I_ and VE_C_. In this example, VE_I_ is 0.7. However, since the vaccine also increases the probability of symptomatic disease by a factor of 3.3, VE_C_ is 0.

To explore the agreement between VE_C_ and VE_I_ under different assumptions of the impact of prior heterologous immunity on the probability of symptomatic disease, we developed an analytical framework (details available in Text S1). Our results suggest that VE_C_ often leads to large underestimates of VE_I_ but can also lead to overestimates depending upon the tradeoff between preventing infections and inducing immunity that can predispose individuals to a more severe outcome (e.g., clinically apparent instead of asymptomatic disease) (Figure 2). Discarding or moving forward with vaccine candidates purely on the basis of VE_C_ could lead to prematurely abandoning vaccines that could have promising population-level impacts or moving forward with an overly optimistic estimate of a vaccine's impact.

**Figure 2:**
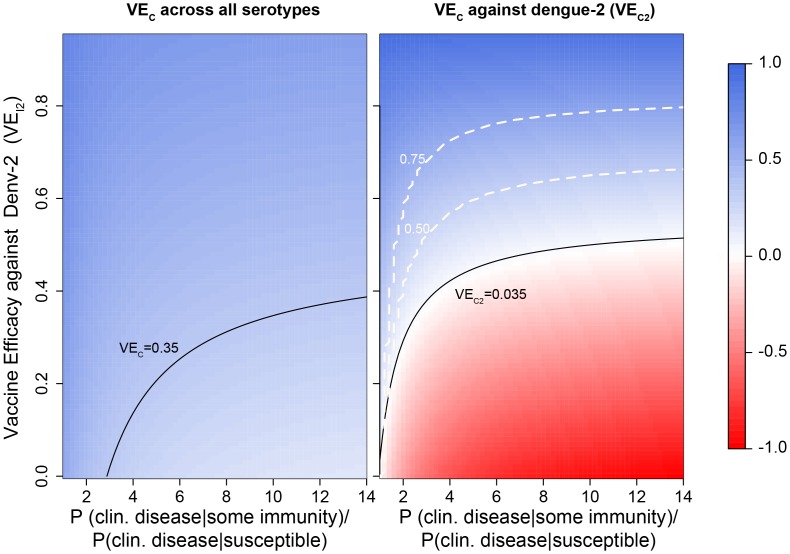
Output from our analytical framework showing the relationship between VE_I_ and VE_C_. For this example, we assumed the following serotype-specific efficacies against infection: VE_I1_ = 0.6 (i.e., VE_I_ for DENV1 = 0.6), VE_I3_ = 0.8, and VE_I4_ = 0.9. We explored a range of VE_I_ against dengue-2 (VE_I2_) (y-axis). The x-axis is the ratio of the probabilities of developing clinical disease in people with and without prior immunity, and hence reflects the extent to which prior heterologous immunity (natural or vaccine acquired) modifies the probability of clinical disease. The background color represents expected vaccine efficacy against clinically apparent infection for all serotypes (left panel) and for dengue-2 (right panel). Solid black contours indicate the clinical vaccine efficacy VE_C_ observed in the Ratchaburi trial [1] according to the intention-to-treat analysis. Dashed white contours indicate levels of the ratio VE_C2_:VE_I2_. Thus, the 0.5 contour divides the regions where VE_C2_ underestimates VE_I2_ by a factor greater (above) or lower (below) than 0.5. In this particular simulation, VE_C_ always underestimates VE_I_.

The discrepancy between VE_C_ and VE_I_ also raises questions about one of the conclusion of this vaccine trial: that the “absence of any sign of disease enhancement…in the presence of non-protective immune responses” to DENV-2 serves as evidence against vaccine-induced enhanced severity of disease. With the inclusion of only clinical outcomes, it is impossible to know what number of individuals in the vaccine or control groups experienced infection and thus what fraction of infections experienced clinical outcomes. Tradeoffs between protective immunity and immunity that may predispose individuals to severe outcome again obscure the results.

While we agree with WHO guidance that clinical disease should be the primary end point of dengue trials and thus VE_C_ the primary measure of vaccines [4], knowledge of infection is critical to correctly interpreting clinical vaccine efficacy results, in particular for diseases like dengue where the relationship between infection and disease is not clear. Furthermore, estimates of the VE_I_ will be necessary to determine the proportion of the population that needs to be vaccinated in order to control transmission. We acknowledge that measures of infection (as measured by virological surveillance among those not experiencing clinically apparent infection, seroconversions, and/or changes in serological responses over time in those not experiencing clinically apparent disease) are imperfect and resource intensive, but surveillance for infection has been performed routinely in other cohort studies [5]. Design of current and future dengue vaccine trials should incorporate additional outcomes in order to fully characterize vaccine candidates given the complexities of the natural history of dengue.

## Supporting Information

Text S1 Description of the analytical framework developed to explore the agreement between VE_C_ and VE_I_.
(PDF)
