# Supplementary material for: Challenges in the Interpretation of Dengue Vaccine Trial Results
Source: PLoS Negl Trop Dis. 2013 Aug 29;7(8):e2126. doi: 10.1371/journal.pntd.0002126 (PMC3757058; doi:10.1371/journal.pntd.0002126)
Supplement: Text S1 — Description of the analytical framework developed to explore the agreement between VEC and VEI. (PDF) [file pntd.0002126.s001.pdf]

Supplementary Material

Calculation of the Vaccine Efficacy against Infection and against Clinical Infection

Here, we describe our probabilistic mathematical framework in detail and state all of our assumptions explicitly. The focus of our investigation is to distinguish between the vaccine efficacy against infection (VE_I) and vaccine efficacy against clinically apparent infection (VE_C). The framework assumes: (i) both groups have identical immune profiles before the vaccine is applied; (ii) individuals can only undergo up to two natural infections by different serotypes and are afterwards immune against further infections.(iii) the control and vaccine groups are embedded in a much larger population so that both groups are exposed to the same force of infection with respect to each serotype, during the course of the study. Thus, the force of infection is not affected by the level of immunity obtained by the vaccine group. (iv) The probability of displaying symptoms upon infection depends solely on prior immunity (regardless of whether the immunity is from infections or vaccination) and on the infecting serotype.

The vaccine efficacy against an infection with serotype i is defined as $V_{Ei} = (I_{TCi} - I_{TVi})/I_{TCi}$, where I_{TCi} is the total incidence (i.e., arising from primary, secondary, etc. infections) for serotype i in the control group and I_{TVi} is the total incidence in the vaccinated group. In contrast, the clinical vaccine efficacy against serotype i is: $VE_{Ci} = (CI_{TCi} - CI_{TVi})/CI_{TCi}$, where CI_{TCi} is the total clinical incidence for serotype i among the controls and CI_{TVi} is the total clinical incidence among the vaccinated group. In what follows, we calculate V_{Ei} and VE_{Ci} assuming the following known quantities: (i) the populations of both groups have a fully susceptible fraction S and a monotypically immune fraction R_i with respect to serotype i, before the application of vaccines. (ii) The force of infection of serotype i acting on the two groups is λ_i. (iii) The probability that a j-th infection with serotype i is clinically apparent is $\chi_j^{(i)}$. (iv) For any individual vaccinee, the efficacy of the vaccine with respect to serotype i is V_{Ei}.

In the control group, the incidence of infections with serotype i arises exclusively from individuals undergoing primary or secondary exposures with values:

$$I_{Ci\cdots} = \lambda_i S, \tag{1a}$$

$$I_{Cji\cdot\cdot} = \lambda_i R_j, \tag{1b}$$

respectively. The first subindex in the secondary incidence above indicates that the primary infection of these individuals was with serotype j ($j \neq i$).

In the vaccine group, primary infections can only occur in individuals that haven't experienced natural infections and failed to seroconvert to the four vaccine serotypes. Thus, the incidence of primary infections with serotype i is

$$I_{Vi\cdots} = \lambda_i S \prod_{j=1}^{4} (1 - V_{Ej}), \tag{2}$$

where the product $\prod_{j=1}^{4}(1-V_{Ej})$ is the fraction of vaccinees that obtained no immunity whatsoever. The expressions for secondary, tertiary and quaternary infections grow quickly in complexity. The incidence of secondary infections (with a primary infection with serotype j) is formed by two contributions

$$I_{Vji\cdot\cdot} = \lambda_i \left(SV_{Ej} \prod_{\substack{k=1 \\ k\neq j}}^{4} (1-V_{Ek}) + R_j \prod_{\substack{k=1 \\ k\neq j}}^{4} (1-V_{Ek}) \right). \tag{3}$$

The first term is the contribution from individuals that obtained monotypical immunity from the vaccine; the second term is the contribution from individuals that suffered a previous infection with serotype j and for whom the vaccine provided no immunity to a serotype other than j.

Analogously to the incidence of secondary infections, the incidence of tertiary infections (with previous viral challenges by serotypes j and k, ordered so that $j<k$) is formed by several terms

$$I_{Vkji\cdot} = \lambda_i \left(SV_{Ek}V_{Ej} \prod_{\substack{l=1 \\ l\neq j,k}}^{4} (1-V_{El}) + (R_kV_{Ej} + R_jV_{Ek}) \prod_{\substack{l=1 \\ l\neq j,k}}^{4} (1-V_{El}) \right) \tag{4}$$

The first term arises from individuals that obtained protection against serotypes j and k from a vaccine; the second is the contribution from individuals whose protection against the two serotypes j and k comes from a previous infection with one of them and from vaccination, to the other. These two possibilities are not permutable and hence they give rise to the two distinct terms $R_kV_{Ej} + R_jV_{Ek}$.

Finally, the incidence of quaternary infections (with previous viral challenges by serotypes j, k and l, ordered so that $j<k<l$) is equal to

$$\begin{aligned} I_{Vlkji} = \lambda_i \Bigg(& SV_{Eell}V_{Ek}V_{Ej}(1-V_{Ei}) + \Big\{ R_lV_{Ek}V_{Ej} \\ & + R_kV_{Ej}V_{El} + R_jV_{El}V_{Ek} \Big\} (1-V_{Ei}) \Bigg), \end{aligned} \tag{5}$$

where the first term accounts for individuals that obtained protection against three serotypes exclusively from vaccination; the terms that follow represent the three possible ways that an individual may posses protection against three serotypes, such that the protection against one arises from a previous natural infection and the protection against the two others arises from the vaccine.

Putting everything together, the total incidences of infection and of clinically

apparent infection with serotype i in the control group are then

$$I_{TCi} = I_{Ci\cdots} + \sum_{j\neq i} I_{Cji\cdot\cdot}, \tag{6a}$$

$$CI_{TCi} = \chi_1^{(i)} I_{Ci\cdots} + \chi_2^{(i)} \sum_{j\neq i} I_{Cji\cdot\cdot}\ . \tag{6b}$$

In the vaccine group, the total incidences of infection and of clinical infection with serotype i are

$$I_{TVi} = I_{Vi\cdots} + \sum_{j\neq i} I_{Vji\cdot\cdot} + \sum_{\substack{j<k \\ j,k\neq i}} I_{Vkji\cdot} + \sum_{\substack{j<k<l \\ j,k,l\neq i}} I_{Vlkji}, \tag{7a}$$

$$CI_{TVi} = \chi_1^{(i)} I_{Vi\cdots} + \chi_2^{(i)} \sum_{j\neq i} I_{Vji\cdot\cdot} + \chi_3^{(i)} \sum_{\substack{j<k \\ j,k\neq i}} I_{Vkji\cdot} + \chi_4^{(i)} \sum_{\substack{j<k<l \\ j,k,l\neq i}} I_{Vlkji}, \tag{7b}$$

respectively. By using eqs. (6) and (7) we obtain the desired expressions for V_{Ei} and VE_{Ci}.

Parameter Values

The values for the parameters utilized in our mathematical framework are as follows. The susceptible and monotypically recovered fractions take on the values $S = 0.1074$ and $R_i = 0.0308$, which are obtained by solving an SIR dengue fever model to steady state, assuming an $R_0 \simeq 5$. Similarly, the force of infection has the value $\lambda_i = 0.0415$, obtained from the steady state of the model just mentioned. The values that we use for the vaccine efficacies V_E are the ones reported in the Ratchaburi trial **??** and are given in Table 1.

DENV1	DENV2	DENV3	DENV4
0.6	variable	0.8	0.9

Table 1: Vaccine Efficacies by Serotype.

Finally, the values for the probabilities that the different infections result in clinical disease ($\chi_j^{(i)}$) are not known with very good precision. We assumed that 5% of primary infections result in clinical disease (regardless of the infecting serotype), and varied the probability of clinical disease in people with prior immunity to one or more serotypes.
